# Involvement of toll-like receptor 9 polymorphism in cervical cancer development

**DOI:** 10.1007/s11033-012-1695-8

**Published:** 2012-06-20

**Authors:** Andrzej Roszak, Margarita Lianeri, Anna Sowińska, Pawel P. Jagodziński

**Affiliations:** 1 Department of Radiotherapy and Gynecological Oncology, Greater Poland Cancer Center, Poznan, Poland; 2 Department of Electroradiology, Poznan University of Medical Sciences, Poznan, Poland; 3 Department of Biochemistry and Molecular Biology, Poznan University of Medical Sciences, 6 Święcickiego St, 60-781 Poznan, Poland; 4 Department of Computer Science and Statistics, Poznan University of Medical Sciences, Poznan, Poland

**Keywords:** Cervical carcinoma, *TLR9*, Polymorphisms

## Abstract

The role played by the polymorphism located in *Toll*-*like Receptor 9* (*TLR9*) as a risk factor of cervical cancer remains elusive. Therefore, we studied the association of the *TLR9* −1486 T/C (rs187084) and C2848T (rs352140) polymorphisms with cervical cancer. The *TLR9* −1486 T/C and C2848T polymorphism was genotyped in 426 patients and 460 unrelated healthy females from the Polish population. Logistic regression analysis adjusting for age, pregnancy, oral contraceptive use, tobacco smoking, and menopausal status showed that both the *TLR9* −1486 T/C and C2848T polymorphisms could be a genetic risk factor for cervical cancer. For the *TLR9* −1486 T/C polymorphism, the adjusted OR for patients with the C/T genotype versus T/T genotype was 1.371 (95 % CI 1.021–1.842, *p* = 0.0361), the adjusted OR for the C/C genotype vs the T/T genotype was 1.300 (95 % CI 1.016–1.507, *p* = 0.0096), and the adjusted OR for the C/T or C/C genotype vs the T/T genotype was 1.448 (95 % CI 1.099–1.908, *p* = 0.0083). For the C2848T polymorphism, the adjusted OR for patients with the C/T genotype vs C/C genotype was 1.443 (95 % CI 1.019–2.043, *p* = 0.0380), the adjusted OR for the T/T genotype vs the C/C genotype was 1.237 (95 % CI 1.016–1.507, *p* = 0.0328), and the adjusted OR for the T/C or T/T genotype vs the C/C genotype was 1.345 (95 % CI 0.976–1.855, *p* = 0.0700). Our studies suggest that the *TLR9* −1486 T/C and C2848T polymorphisms may be a genetic risk factor for cervical cancer.

## Introduction

Cervical carcinogenesis is a multi-step process associated with refractory infection by high-risk human papillomavirus (HPV) types [1, 2]. This includes the transformation of normal cervical epithelium to cervical intraepithelial neoplasia (CIN), which is transformed to invasive cervical carcinoma [1, 2]. The HPV oncoproteins E6 and E7 play a key role in cervical carcinogenesis via the disturbance of apoptosis, the cell cycle, and adaptive immune surveillance [3]. Only a minority of HPV infected women will develop CIN or cervical cancer, suggesting that HPV is not a sufficient separate factor responsible for tumorigenesis of the cervix [4]. Many epidemiological studies have also indicated that environmental factors, contraceptive use, smoking, and genetic factors may also contribute to cervical carcinogenesis [5–9].

Recently, several studies have demonstrated that HPV infections may change the expression of toll-like receptors (TLRs), which interferes with TLRs signaling during early and late malignant transformation of the cervix [10–13]. TLRs recognize exogenous pathogen-associated molecular patterns and they play an elementary role in the innate immune response [14].

In humans, the family of TLRs includes ten members, which can be divided based on their localization in the cell [14]. TLR1, TLR2, TLR4, TLR5, TLR6, and TLR10 are usually situated on the cell surface, whereas TLR3, TLR7, TLR8, and TLR9 are localized almost exclusively in endosomes [14–17]. TLR9 have been shown to recognize unmethylated cytosine–phosphate–guanine (CpG) dinucleotide motifs located in bacterial, viral and fungal DNA [17, 18]. TLR9 is present in macrophages, dendritic cells and intestinal epithelium, as well as respiratory epithelial and keratinocytes cells [ 16, 17, 19–21 ]. It has been demonstrated that the −1486 T/C (rs187084), −1237T/C (rs5743836) and C2848T (rs352140) polymorphisms of *TLR9* located in the same block of linkage disequilibrium (LD) may change *TLR9* expression (HapMap CEU data http://hapmap.ncbi.nlm.nih.gov/) [22–24].

There are two studies on the association of the *TLR9* 1486 T/C (rs187084) and C2848T (rs352140) polymorphisms with cervical cancer in various populations, however, the results are inconsistent [25, 26]. Therefore, we aimed to study whether *TLR9* −1486 T/C (rs187084) and C2848T (rs352140) can be a genetic risk factor of cervical cancer in the Polish population.

## Patients and methods

### Patients and controls

The patient group was composed of 426 women with histologically determined cervical carcinoma according to the International Federation of Gynecology and Obstetrics (FIGO). All women were enrolled between April 2007 and July 2011 at the Department of Radiotherapy, Greater Poland Cancer Center in Poznan, Poland (Table 1). The controls included four hundred sixty unrelated healthy female volunteers who were matched by age to the patients (Table 1). Information about pregnancy, oral contraceptive use, tobacco smoking, and menopausal status was obtained as part of the patient history. Patients and controls were Caucasian, enrolled from the Wielkopolska area of Poland. All subjects provided written informed consent. The study was approved by the Local Ethical Committee of Poznan University of Medical Sciences.

**Table 1:** Clinical and demographic characteristics of patients and controls

Characteristic	Patients (*n* = 426) (%)	Controls (*n* = 460) (%)
^a^Mean age (years) ± SD	51.8 ± 9.7	51.9 ± 9.8
Tumor stage		
IA	55 (12.9)	
IB	57 (13.4)	
IIA	56 (13.1)	
IIB	47 (11.0)	
IIIA	143 (33.6)	
IIIB	54 (12.7)	
IVA	8 (1.9)	
IVB	6 (1.4)	
Histological grade		
G1	77 (18.1)	
G2	133 (31.2)	
G3	94 (22.1)	
Gx	122 (28.6)	
Histological type		
Squamous cell carcinoma	361 (84.8)	
Adenocarcinoma	50 (11.7)	
Other	15 (3.5)	
Pregnancy		
Never	45 (10.6)	51 (11.1)
Ever	381 (89.4)	409 (88.9)
Oral contraceptive pill use		
Never	234 (54.9)	257 (55.9)
Ever	192 (45.1)	203 (44.1)
Tobacco smoking		
Never	278 (65.3)	312 (67.8)
Ever	148 (34.7)	148 (32.2)
Menopausal status		
Premenopausal	149 (35.0)	175 (41.1)
Postmenopausal	277 (65.0)	285 (61.9)

^a^Age at first diagnosis

### Genotyping

DNA was obtained from peripheral leucocytes using a standard salting out procedure. Identification of the *TLR9* -1486 T/C (rs187084) and C2848T (rs352140) polymorphic variant was performed by polymerase chain reaction-restriction fragment length polymorphism (PCR–RFLP). PCR was conducted employing primer pair 5′TTCATTCATTCAGCCTTCACTCA 3′, 5′ GAGTCAAAGCCACAGTCCACA 3′ and 5′ GCAGCACCCTCAACTTCACC 3′and 5′ GGCTGTGGATGTTGTTGTGG 3′, respectively.

The PCR-amplified fragments 565 bp in length bearing the *TLR9* -1486 T/C (rs187084) polymorphism were digested with restriction enzyme Afl II (C/TTAAG) New England BioLabs, (Ipswich, USA). The *TLR9* T allele was cleaved into 416 and 149 bp fragments, whereas the *TLR9* C allele remained uncut. The PCR-amplified fragments 360 bp in length corresponding to the *TLR9* C2848T polymorphism were subjected to digestion with the endonuclease BstUI (CG/CG) New England BioLabs, (Ipswich, USA). The *TLR9* C allele was cleaved into 227 and 133 bp fragments, whereas the *TLR9* T allele remained uncut. DNA fragments were separated by electrophoresis on 3 % agarose gel and visualized by ethidium bromide staining. The *TLR9* 1486 T/C and C2848T polymorphism was confirmed by repeated PCR–RFLP. Moreover, the restriction analysis was confirmed by commercial sequencing analysis.

### Statistical analysis

The distribution of genotypes in patients and controls was examined for deviation from Hardy–Weinberg equilibrium using exact and log likelihood ratio χ^2^ tests [http://ihg.gsf.de/cgi-bin/hw/hwa1.pl]. The polymorphism was tested for association with cervical cancer using the χ^2^ test for trend (p_trend_). The χ^2^ test was employed to examine differences in genotypic and allelic distribution between patients and controls. The odds ratio (OR) and 95 % confidence intervals (95 % CI) were calculated. Unconditional logistic regression analysis was used to adjust for the effect of confounders such as age, pregnancy, oral contraceptive use, tobacco smoking, and menopausal status. A *p* value of <0.05 was considered statistically significant.

## Results

### Prevalence of the *TLR9* −1486 T/C polymorphism in women with cervical cancer

There was a higher frequency of the *TLR9* CC genotype in women with cervical cancer compared to healthy individuals, which was 0.19 and 0.14, respectively (Table 2). We also found increased *TLR9* C/T heterozygote frequency in patients than in controls, which was 0.48 and 0.44, respectively (Table 2). There was also an increased *TLR9* C allele frequency in patients than in controls, which was 0.43 and 0.36, respectively (Table 2). The p value of the χ^2^ test of the trend observed for the *TLR9 −1486 T/C* polymorphism was statistically significant (p_trend_ = 0.0042). Logistic regression analysis showed a significant contribution of the *TLR9 −*1486 T/C polymorphism to cervical cancer (Table 2). The adjusted OR for patients with the C/T genotype vs T/T genotype was 1.371 (95 % CI 1.021–1.842, *p* = 0.0361), the adjusted OR for the C/C genotype vs the T/T genotype was 1.300 (95 % CI 1.016–1.507, *p* = 0.0096), and the adjusted OR for the C/T or C/C genotype vs the T/T genotype was 1.448 (95 % CI 1.099–1.908, *p* = 0.0083) (Table 2).

**Table 2:** Contribution of the *TLR9 −*1486 T/C (rs187084) and C2848T (rs352140) polymorphisms to cervical cancer

Polymorphism	rs no.	Genotype	Patients (frequency)	Controls (frequency)	Odds ratio (95 % CI)	*p* ^a^	Adjusted odds ratio (95 % CI)^b^	*p* ^a^
**−1486 T/C**	rs187084	TT	141 (0.33)	193 (0.42)	Referent	–	Referent	–
		CT	206 (0.48)	203 (0.44)	1.389 (1.038–1.858)	0.0267	**1.371 (1.021–1.842)**	**0.0361**
		CC	79 (0.19)	64 (0.14)	**1.690 (1.138–2.508)**	**0.0089**	**1.300 (1.016–1.507)**	**0.0096**
		CT + CC	285 (0.67)	267 (0.58)	**1.461 (1.111–1.922)**	**0.0066**	**1.448 (1.099–1.908)**	**0.0083**
	Minor allele frequency		0.43	0.36	**1.327 (1.096–1.607)**	**0.0037**		
**C2848T**	rs352140	CC	87 (0.20)	122 (0.27)	Referent	–	Referent	–
		CT	230 (0.54)	235 (0.51)	1.372 (0.987–1.909)	0.0594	**1.443 (1.019–2.043)**	**0.0380**
		TT	109 (0.26)	103 (0.22)	**1.484 (1.010–2.181)**	**0.0441**	**1.237 (1.016–1.507)**	**0.0328**
		CT + TT	339 (0.80)	338 (0.73)	**1.406 (1.028–1.925)**	**0.0326**	1.345 (0.976–1.855)	0.0700
	Minor allele frequency		0.53	0.48	1.204 (0.999–1.452)	0.0506		

Significant results are highlighted in bold font

^a^χ^2^ analysis

^b^ORs were adjusted by age, pregnancy, oral contraceptive use, tobacco smoking, and menopausal status

### Prevalence of the *TLR9* C2848T polymorphism in women with cervical cancer

We observed an increased frequency of the *TLR9* TT genotype in patients than controls, which was 0.26 and 0.22, respectively (Table 2). The *TLR9* C/T heterozygote frequency in women with cervical cancer was also increased compared to healthy individuals and amounted to 0.54 and 0.51, respectively (Table 2). We also observed a higher *TLR9* T allele frequency in patients then healthy individuals, which was 0.53 and 0.48, respectively (Table 2). The p value of the χ^2^ test of the trend observed for the *TLR9* C2848T polymorphism was statistically significant (p_trend_ = 0.0449). Logistic regression analysis showed a significant association of the *TLR9* C2848T polymorphism with cervical cancer (Table 2). The adjusted OR for patients with the C/T genotype vs C/C genotype was 1.443 (95 % CI 1.019–2.043, *p* = 0.0380), the adjusted OR for the T/T genotype vs the C/C genotype was 1.237 (95 % CI 1.016–1.507, *p* = 0.0328), and the adjusted OR for the T/C or T/T genotype vs the C/C genotype was 1.345 (95 % CI 0.976–1.855, *p* = 0.0700) (Table 2).

## Discussion

TLR9 plays a crucial role in pathogen recognition and activation of innate immunity [15, 18]. Stimulation of TLR9 activates human B cells and plasmacytoid dendritic cells, causing T helper-1 type immune responses and antitumor responses [18, 27–31]. The role of the TLR9 pathway in anticancer treatment has been considered in animal models and patients with renal carcinoma, malignant melanoma, and non-Hodgkin’s lymphoma [27–31]. Moreover, the significance of the TLR9 pathway has been demonstrated in vaccine treatment of cervical carcinoma in murine model [28]. Mansilla et al. [28] demonstrated that intratumoral administration of EDA-HPVE7 fusion protein in combination with TLR9 ligand CpG-B was able to eradicate large established cervical tumors.

HPV infects primitive basal keratinocytes; however, its abundant expression and viral assembly take place only in the upper layers of the stratum spinosum and granulosum of squamous epithelia [32]. Keratinocytes bear TLR9, and stimulation of TLR9 may result in the production of a spectrum of mediators influencing the function of immune cells [21]. Some studies have suggested that changes occur in *TLR9* expression during cervical carcinogenesis [11, 13]. Recently, Hasimu et al. (2007) found that the expression of *TLR9* can be up-regulated by HPV16 infection in CIN and in cervical squamous carcinoma cells [11]. They also suggested that TLR9 may play important roles in the development and progression of CIN and cervical carcinoma [11]. Adding to the above findings are those of Hasan et al. [13], who demonstrated that HPV16 infection of human primary keratinocytes reduced *TLR9* transcription and lead to a functional loss of TLR9-regulated pathways. This may suggest that polymorphisms of *TLR9* that modulate the expression of *TLR9* may have an effect on the etiopathogenesis of cervical cancer [33].

We observed a contribution of the *TLR9* −1486 T/C (rs187084) and C2848T (rs352140) polymorphisms to the risk of cervical cancer in a Polish population. Recent studies conducted by Chen et al. [25] demonstrated that the *TLR9* −1486 T/C (rs187084) polymorphism, located in the LD block with rs352140, was associated with a significantly increased risk of cervical cancer. In contrast, Pandey et al. [26] showed that the TT genotype of *TLR9* (rs352140) displayed borderline significance in increased risk for advanced cervical cancer in a North India population.

These differences in the effect of *TRL9* polymorphism on the susceptibility to cervical cancer development between our and Hindu populations may result from racial heterogeneity, the size of the studied groups, and the action of distinct behavioral and environmental factors [33].

To date, the *TLR9* C2848T (rs352140) polymorphism has also been associated with Hodgkin’s lymphoma, periodontitis, ulcerative colitis, and systemic lupus erythematosus [34–37]. The other *TLR9* variants have also been found to be risk factors of atopic eczema, tuberculosis, *Helicobacter pylori*-induced gastritis, and rheumatoid arthritis [38–41]. Moreover, these *TLR9* variants may influence the clinical course of HIV-1 infection, and the development of endometrial cancer, osteoarthritis, and non-Hodgkin lymphoma [42–45].

The role of the rs352140 polymorphism on *TLR9* expression has been suggested by Kikuchi et al. [24],who demonstrated that the *TLR9* 2848 TT genotype was associated with a higher expression of *TLR9* and an increased frequency of IgM + B cells. It has been believed that chronic inflammation may lead to cancer development and progression (33). The increased expression of the *TLR9* 2848 T variant in precursor malignant lesion cells combined with infection by various pathogens might support inflammation and cervical cancer development (33).

Our studies suggest that the C2848T (rs352140) polymorphism might be a risk factor of cervical cancer in Polish women. Our genetic evaluation is the first in a Caucasian cohort; therefore this study should be replicated in a larger and independent cohort.

## References

[1] Walboomers JM, Jacobs MV, Manos MM (1999). Human papillomavirus is a necessary cause of invasive cervical cancer worldwide. J Pathol.

[2] Muñoz N, Bosch FX, de Sanjosé S, Herrero R, Castellsagué X, Shah KV (2003). Epidemiologic classification of human papillomavirus types associated with cervical cancer. N Engl J Med.

[3] Münger K, Howley PM (2002). Human papillomavirus immortalization and transformation functions. Virus Res.

[4] Giuliano AR, Harris R, Sedjo RL, Baldwin S, Roe D, Papenfuss MR (2002). Incidence, prevalence, and clearance of type-specific human papillomavirus infections: the Young Women’s Health Study. J Infect Dis.

[5] Magnusson PK, Sparen P, Gyllensten UB (1999). Genetic link to cervical tumours. Nature.

[6] Castellsague X, Munoz N (2003) Chap. 3: Cofactors in human papillomavirus carcinogenesis–role of parity, oral contraceptives, and tobacco smoking. J Natl Cancer Inst Monogr 20–28 12807941

[7] Magnusson PK, Lichtenstein P, Gyllensten UB (2000). Heritability of cervical tumours. Int J Cancer.

[8] Hemminki K, Chen B (2006). Familial risks for cervical tumors in full and half siblings: etiologic apportioning. Cancer Epidemiol Biomarkers Prev.

[9] Moreno V, Bosch FX, Muñoz N, Meijer CJ, Shah KV, Walboomers JM (2002). Effect of oral contraceptives on risk of cervical cancer in women with human papillomavirus infection: the IARC multicentric case-control study. Lancet.

[10] DeCarlo CA, Rosa B, Jackson R, Niccoli S, Escott NG, Zehbe I (2012). Toll-like receptor transcriptome in the HPV-positive cervical cancer microenvironment. Clin Dev Immunol.

[11] Hasimu A, Ge L, Li QZ, Zhang RP, Guo X (2011). Expressions of Toll-like receptors 3, 4, 7, and 9 in cervical lesions and their correlation with HPV16 infection in Uighur women. Chin J Cancer.

[12] Kim WY, Lee JW, Choi JJ, Choi CH, Kim TJ, Kim BG (2008). Increased expression of Toll-like receptor 5 during progression of cervical neoplasia. Int J Gynecol Cancer.

[13] Hasan UA, Bates E, Takeshita F, Biliato A, Accardi R, Bouvard V (2007). TLR9 expression and function is abolished by the cervical cancer-associated human papillomavirus type 16. J Immunol.

[14] Akira S, Takeda K (2004). Toll-like receptor signalling. Nat Rev Immunol.

[15] Ahmad-Nejad P, Häcker H, Rutz M, Bauer S, Vabulas RM, Wagner H (2002). Bacterial CpG-DNA and lipopolysaccharides activate Toll-like receptors at distinct cellular compartments. Eur J Immunol.

[16] Heil F, Ahmad-Nejad P, Hemmi H, Hochrein H, Ampenberger F, Gellert T (2003). The Toll-like receptor 7 (TLR7)-specific stimulus loxoribine uncovers a strong relationship within the TLR7, 8 and 9 subfamily. Eur J Immunol.

[17] Latz E, Schoenemeyer A, Visintin A, Fitzgerald KA, Monks BG, Knetter CF (2004). TLR9 signals after translocating from the ER to CpG DNA in the lysosome. Nat Immunol.

[18] Hemmi H, Takeuchi O, Kawai T, Kaisho T, Sato S, Sanjo H (2000). A Toll-like receptor recognizes bacterial DNA. Nature.

[19] Schmausser B, Andrulis M, Endrich S, Lee SK, Josenhans C, Müller-Hermelink HK (2004). Expression and subcellular distribution of toll-like receptors TLR4, TLR5 and TLR9 on the gastric epithelium in *Helicobacter pylori* infection. Clin Exp Immunol.

[20] Akhtar M, Watson JL, Nazli A, McKay DM (2003). Bacterial DNA evokes epithelial IL-8 production by a MAPK-dependent, NF-kappaB-independent pathway. FASEB J.

[21] Sugita K, Kabashima K, Atarashi K, Shimauchi T, Kobayashi M, Tokura Y (2007). Innate immunity mediated by epidermal keratinocytes promotes acquired immunity involving Langerhans cells and T cells in the skin. Clin Exp Immunol.

[22] Tao K, Fujii M, Tsukumo S, Maekawa Y, Kishihara K, Kimoto Y (2007). Genetic variations of Toll-like receptor 9 predispose to systemic lupus erythematosus in Japanese population. Ann Rheum Dis.

[23] Lange NE, Zhou X, Lasky-Su J, Himes BE, Lazarus R, Soto-Quirós M (2011). Comprehensive genetic assessment of a functional TLR9 promoter polymorphism: no replicable association with asthma or asthma-related phenotypes. BMC Med Genet.

[24] Kikuchi K, Lian ZX, Kimura Y, Selmi C, Yang GX, Gordon SC (2005). Genetic polymorphisms of toll-like receptor 9 influence the immune response to CpG and contribute to hyper-IgM in primary biliary cirrhosis. J Autoimmun.

[25] Chen X, Wang S, Liu L, Chen Z, Qiang F, Kan Y et al. (2011) A genetic variant in the promoter region of toll-like receptor 9 and cervical cancer susceptibility. DNA Cell Biol. doi:10.1089/dna.2011.1427 10.1089/dna.2011.1427 22059466

[26] Pandey S, Mittal B, Srivastava M, Singh S, Srivastava K, Lal P (2011). Evaluation of Toll-like receptors 3 (c.1377C/T) and 9 (G2848A) gene polymorphisms in cervical cancer susceptibility. Mol Biol Rep.

[27] Carpentier AF, Chen L, Maltonti F, Delattre JY (1999). Oligodeoxynucleotides containing CpG motifs can induce rejection of a neuroblastoma in mice. Cancer Res.

[28] Mansilla C, Berraondo P, Durantez M, Martínez M, Casares N, Arribillaga L et al. (2011) Eradication of large tumors expressing human papillomavirus E7 protein by therapeutic vaccination with E7 fused to the extra domain a from fibronectin. Int J Cancer. doi: 10.1002/ijc.26412 10.1002/ijc.26412 21898393

[29] Hofmann MA, Kors C, Audring H, Walden P, Sterry W, Trefzer U (2008). Phase 1 evaluation of intralesionally injected TLR9-agonist PF-3512676 in patients with basal cell carcinoma or metastatic melanoma. J Immunother.

[30] Leonard JP, Link BK, Emmanouilides C, Gregory SA, Weisdorf D, Andrey J (2007). Phase I trial of toll-like receptor 9 agonist PF-3512676 with and following rituximab in patients with recurrent indolent and aggressive non Hodgkin’s lymphoma. Clin Cancer Res.

[31] Thompson JA, Kuzel T, Drucker BJ, Urba WJ, Bukowski RM (2009). Safety and efficacy of PF-3512676 for the treatment of stage IV renal cell carcinoma: an open-label, multicenter phase I/II study. Clin Genitourin Cancer.

[32] Doorbar J (2005). The papillomavirus life cycle. J Clin Virol.

[33] El-Omar EM, Ng MT, Hold GL (2008). Polymorphisms in Toll-like receptor genes and risk of cancer. Oncogene.

[34] Holla LI, Vokurka J, Hrdlickova B, Augustin P, Fassmann A (2010). Association of Toll-like receptor 9 haplotypes with chronic periodontitis in Czech population. J Clin Periodontol.

[35] Fuse K, Katakura K, Sakamoto N, Ohira H (2010). Toll-like receptor 9 gene mutations and polymorphisms in Japanese ulcerative colitis patients. World J Gastroenterol.

[36] Mollaki V, Georgiadis T, Tassidou A, Ioannou M, Daniil Z, Koutsokera A (2009). Polymorphisms and haplotypes in TLR9 and MYD88 are associated with the development of Hodgkin’s lymphoma: a candidate-gene association study. J Hum Genet.

[37] Xu CJ, Zhang WH, Pan HF, Li XP, Xu JH, Ye DQ (2009). Association study of a single nucleotide polymorphism in the exon 2 region of toll-like receptor 9 (TLR9) gene with susceptibility to systemic lupus erythematosus among Chinese. Mol Biol Rep.

[38] Novak N, Yu CF, Bussmann C, Maintz L, Peng WM, Hart J (2007). Putative association of a TLR9 promoter polymorphism with atopic eczema. Allergy.

[39] Velez DR, Wejse C, Stryjewski ME, Abbate E, Hulme WF, Myers JL (2010). Variants in toll-like receptors 2 and 9 influence susceptibility to pulmonary tuberculosis in Caucasians, African-Americans, and West Africans. Hum Genet.

[40] Ng MT, Van’t Hof R, Crockett JC, Hope ME, Berry S, Thomson J (2010). Increase in NF-kappaB binding affinity of the variant C allele of the toll-like receptor 9–1237T/C polymorphism is associated with Helicobacter pylori-induced gastric disease. Infect Immun.

[41] Etem EO, Elyas H, Ozgocmen S, Yıldırım A, Godekmerdan A (2011). The investigation of toll-like receptor 3, 9 and 10 gene polymorphisms in Turkish rheumatoid arthritis patients. Rheumatol Int.

[42] Bochud PY, Hersberger M, Taffé P, Bochud M, Stein CM, Rodrigues SD (2007). Polymorphisms in Toll-like receptor 9 influence the clinical course of HIV-1 infection. AIDS.

[43] Ashton KA, Proietto A, Otton G, Symonds I, McEvoy M, Attia J (2010). Toll-like receptor (TLR) and nucleosome-binding oligomerization domain (NOD) gene polymorphisms and endometrial cancer risk. BMC Cancer.

[44] Su SL, Yang HY, Lee CH, Huang GS, Salter DM, Lee HS (2012). The (-1486T/C) promoter polymorphism of the TLR-9 gene is associated with end-stage knee osteoarthritis in a Chinese population. J Orthop Res.

[45] Carvalho A, Cunha C, Almeida AJ, Osório NS, Saraiva M, Teixeira-Coelho M et al. (2011) The rs5743836 polymorphism in TLR9 confers a population-based increased risk of non-Hodgkin lymphoma. Genes Immun. doi:10.1038/gene.2011.59 10.1038/gene.2011.59 PMC3876733 21866115

